# Guiding Efficient, Effective, and Patient-Oriented Electrolyte Replacement in Critical Care: An Artificial Intelligence Reinforcement Learning Approach

**DOI:** 10.3390/jpm12050661

**Published:** 2022-04-20

**Authors:** Niranjani Prasad, Aishwarya Mandyam, Corey Chivers, Michael Draugelis, C. William Hanson, Barbara E. Engelhardt, Krzysztof Laudanski

**Affiliations:** 1 Department of Computer Science, Princeton University, Princeton, NJ 08540, USA; prasad.niranjani@gmail.com (N.P.); amandyam@princeton.edu (A.M.); bee@princeton.edu (B.E.E.); 2 Gladstone Institutes, San Francisco, CA 94158, USA; 3 University of Pennsylvania Health System, Philadelphia, PA 19104, USA; corey.chivers@pennmedicine.upenn.edu (C.C.); michael.draugelis@pennmedicine.upenn.edu (M.D.); hansonb@uphs.upenn.edu (C.W.H.III); 4 Department of Anesthesiology and Critical Care, University of Pennsylvania, Philadelphia, PA 19104, USA; 5 Penn Medicine Predictive Healthcare, University of Pennsylvania Health System, Philadelphia, PA 19104, USA; 6 Leonard Davis Institute of Healthcare Economics, University of Pennsylvania, Philadelphia, PA 19104, USA

**Keywords:** electrolytes, electronic health records, artificial intelligence, machine learning, reinforcement learning, decision support systems, retrospective studies, MIMIC-IV

## Abstract

Both provider- and protocol-driven electrolyte replacement have been linked to the over-prescription of ubiquitous electrolytes. Here, we describe the development and retrospective validation of a data-driven clinical decision support tool that uses reinforcement learning (RL) algorithms to recommend patient-tailored electrolyte replacement policies for ICU patients. We used electronic health records (EHR) data that originated from two institutions (UPHS; MIMIC-IV). The tool uses a set of patient characteristics, such as their physiological and pharmacological state, a pre-defined set of possible repletion actions, and a set of clinical goals to present clinicians with a recommendation for the route and dose of an electrolyte. RL-driven electrolyte repletion substantially reduces the frequency of magnesium and potassium replacements (up to 60%), adjusts the timing of interventions in all three electrolytes considered (potassium, magnesium, and phosphate), and shifts them towards orally administered repletion over intravenous replacement. This shift in recommended treatment limits risk of the potentially harmful effects of over-repletion and implies monetary savings. Overall, the RL-driven electrolyte repletion recommendations reduce excess electrolyte replacements and improve the safety, precision, efficacy, and cost of each electrolyte repletion event, while showing robust performance across patient cohorts and hospital systems.

## 1. Introduction

The process of evaluating clinical data in the intensive care unit (ICU) to make diagnostic or therapeutic decisions is highly demanding, repetitive, and often requires over 100 decisions per day on average per provider [1,2]. This approach is almost always reactive and often not patient-centric [3,4,5,6,7]. The high stakes and pace of ICU operations put a strain on providers, leading to the frequent reliance on cognitive shortcuts [2,6,7,8,9]. Prior experience and legal or ethical expectations further influence clinical decision making, along with the dynamics between different care providers and the availability of personnel, resources, or procedural constraints [10,11]. The delegation of the decision-making process to standardized protocols is often employed with the hope of improving outcomes and reducing variability [12,13]. However, protocols are inherently inflexible and prone to bias in their formulation, often contributing to poor adherence in practice [14]. Their clinical benefits may be overestimated, while the risks or expected frequency of unintended side effects may be underestimated [15,16,17,18]. Taking the aforementioned problems into the context of the current practice of electrolyte replacements, there is a clear need for more data-driven and patient-specific approaches [5].

The management of serum electrolyte levels is omnipresent in the ICU, but they have a narrow therapeutic range. Even small fluctuations outside the reference range may result in severe clinical consequences, for example, cardiac arrest. Electrolyte imbalances arise through the highly complex pathological processes of illness, pre-existing conditions, or administered medications [19,20]. Furthermore, the relationship between the target values of electrolyte levels and their clinical benefit is complex [21]. A provider-directed approach to electrolyte repletion can therefore often lead to unaddressed episodes of low electrolyte levels, high rates of superfluous replacements, and a poorly allocated use of provider time and expenditures, while creating risk to the patient [17,22,23,24].

Artificial intelligence (AI), or machine learning methods, such as reinforcement learning, presents a pathway for adaptive guidance of healthcare delivery; they are well-suited to leverage information from the data-rich ICU environment [25]. The reinforcement learning framework potentially enables the planning and management of patient care within the dynamic processes of critical illness, incorporating both patients’ needs and healthcare workflow constraints. Such a system is well-suited to the data-rich ICU setting, to adjust recommendations based on ever-changing patient characteristics.

Here, we built and retrospectively evaluated an artificial intelligence (AI) engine intended to provide a clinician-in-the-loop decision support system for electrolyte repletion, focusing on the management of potassium, magnesium, and phosphate levels in hospitalized, critically ill patients. To date, machine learning methods have been applied to the closely related problem of fluid resuscitation for management of hypotension in critically ill patients [26,27]. These works suggest that machine learning methods can be used to retrospectively analyze and learn from clinician behavior.

We used reinforcement learning (RL), an AI approach, to address differences between patients’ current and target physiological characteristics in a dynamic way [28,29]. Specifically, we adapted RL methods to govern intravenous potassium, magnesium, and phosphate repletion, with the objective of minimizing variation in electrolyte levels and managing repletion costs. This manuscript describes the formulation and methodology of the RL framework, the data preprocessing and training procedure used, and application in silico with assessment of performance. Finally, we validate the methodology on a second dataset, as implementation of AI in one system may fail when applied to a different setting than the one used for initial training and in the in silico trial.

## 2. Materials and Methods

The Institutional Review Board of the University of Pennsylvania approved this study (#823822).

### 2.1. Dataset and Cohort Selection

The data used in this retrospective study were drawn from electronic health records (EHR) from critical care units between 2010 and 2015 across three major hospitals in the University of Pennsylvania Health System (UPHS). A total of 459,173 unique critical care admissions were made available for analysis.

**Figure 1:**
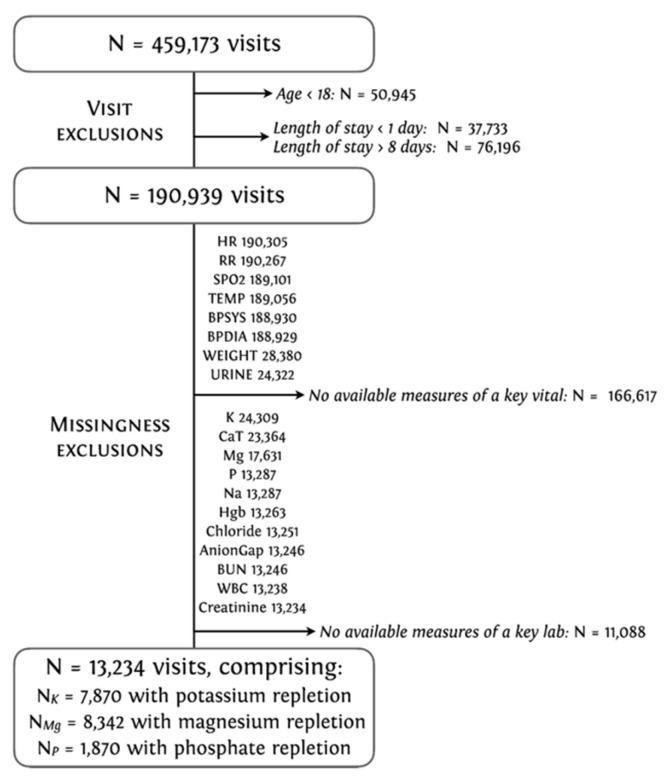
Data cohort selection criteria (demonstrated in the UPHS database). Heart rate, (HR); Respiratory rate, (RR); Oxygen saturation, (SPO_2_); Temperature, (TEMP); Systolic blood pressure (BPSYS); Diastolic blood pressure, (BPDIA).

We extracted three (overlapping) sub-cohorts, selecting for data from all adult patients (over the age of 18) with a hospital visit of a duration between one and eight days from UPHS. We filtered the data to include patients with at least one recorded value of all key vitals and labs (summarized in Table 1), including weight at the time of admission, which was recorded more sparsely in the dataset. A total of 13,234 hospital visits were used, each with a minimum of one instance of either potassium, magnesium, or phosphate repletion over the course of the visit: 7870 with potassium, 8342 with magnesium, and 1768 with phosphate replacements (Figure 1) [30].

Each hospital visit was divided into 6 h intervals to reflect the frequency with which staff may be reasonably able to react to automated recommendations. Clinically nonviable outliers in measured patient vitals and lab values were filtered out, and the mean of remaining measurements within a given six-hour interval was taken as representative of the value at this time step. Missing values were imputed with the last measurement for up to 48 h and otherwise imputed with the population mean value of each lab or vital sign.

### 2.2. Model Framework

The task of electrolyte repletion during patient visits to the ICU was modeled as a Markov decision process (MDP), *M = <S, A, P, R, γ>* [31]. Over a sequence of discrete time steps at 6 h intervals, we observed the patient in some state in *S*, chose a treatment action from set *A*, and observed a stochastic transition to a new patient state (according to probability distribution *P*). Feedback from the transition was in the form of reward *R*. The 6 h interval was chosen to mimic hospital workflow. Our objective was to learn an optimal policy π, mapping from a state in a continuous space *S* to an action in a discrete set *A* that maximizes the total discounted reward collected over the patient visit, where discount factor *γ* determines the relative importance of immediate versus distant rewards. Details of the protocol are included in Appendix A [30].

In defining the clinical condition of the patient in our model, we incorporated a total of 52 factors based on their relevance to or potential influence on electrolyte homeostasis in the patient (Table 1) [19,20]. We also included the administration of intravenous (IV) and oral (PO) electrolytes, and other potentially relevant medications administered over the past 6 h interval. To define the actionable AI events (action space *A*), we allowed for dosage rates in line with standard clinical practice (Table 2). The dosing of these drugs was considered at one of six possible rates: 0–10 mEq/h infused over 1, 2, or 3 h; 10–20 mEq/h over 2, 4, or 6 h, or some combination of both intravenous and oral supplements. Repletion rates and doses were chosen in the same way for magnesium (Mg) and phosphates (P) (Table 2).

The AI performance was guided by: (i) a penalty for electrolyte levels above the reference range, (ii) a penalty for electrolyte levels below this range, (iii) the corresponding effective cost of PO repletion, and (iv) cost of repletion. The AI reward function was a weighted sum of these four conditions relevant to the current patient condition, the immediate action advised by AI, and the next state (Appendix A). The aim of our RL algorithm was learning a policy that would maximize the cumulative reward or, equivalently, minimize the total accumulated penalties over the course of the patient’s admission.

### 2.3. Model Training

Data from the 13,234 hospital visits obtained from the UPHS dataset after applying our exclusion criteria were randomly split into 7000 visits in the training set to learn an optimal repletion policy, and 6164 in the test set to evaluate our learned policy on held-out data. By setting the sampling interval at 6 h and creating one-step transition samples of the form <state, action, reward, next state>, we produced a total of 54,228 samples in the training set for the potassium sub-cohort, 59,775 for magnesium, and 15,863 for phosphate.

Fitted Q-iteration (FQI), a data-efficient algorithm for offline reinforcement learning, was used to learn optimal treatment policies from these sets of patient state transitions [32]. The FQI algorithm learns a Q-value function, which is an estimate of the long-term rewards of each available action at a given patient state from the training data. Then, on our test data, we can use the learned Q-value function to choose the action that maximizes the rewards at a given patient state to identify the optimal treatment policy [33,34].

For each electrolyte repletion task, the learned policy first decides whether to administer a supplement and if so, by what route (oral, intravenous, or both). The second and third steps determine the most appropriate dosage and infusion time for oral or intravenous repletion, respectively. A retrospective off-policy evaluation (OPE) of the learned policy was performed using a frequency analysis of action recommendations, a qualitative analysis of the policy on patient trajectories, and fitted-Q evaluation (FQE), a state-of-the-art approach to estimating the expected accumulated reward of the learned policies [35].

### 2.4. Validation on MIMIC-IV

We extracted 40,000 adult ICU patients from MIMIC-IV to validate our RL algorithm [36]. The data include deidentified hospital patients admitted to one of the critical care units of the Beth Israel Deaconess Medical Center between 2008 and 2019. We used 40,000 unique critical care visits for our validation. As with the UPHS data, we split the visits into 32,000 for the training set and 8000 for the test set to evaluate our learned policy. After filtering, this data yielded a total of 54,228 samples in the training set for the potassium sub-cohort, 59,775 for magnesium, and 15,863 for phosphate. We also followed a similar imputation protocol when the exact value of a lab or vital was unknown. When training our AI algorithm, we used a set of 63 covariates to represent patient state. Our reward function is identical to the one used on the UPHS dataset, where rewards accumulate when the patient is within the reference range for a given electrolyte. 

### 2.5. Financial Modeling

Financial modeling was carried out using the attached workflow, drawing upon prior work (Appendix B) [24]. The salaries were taken from a U.S. job site [37,38]. The prices of the medication were set using the Lexicon [39]. The prices of laboratory tests were obtained from the CMS schedule for the year 2020 [40]. In general, the lowest bracket was applied uniformly where estimates for wages, lab, and salaries were incorporated into the modeling. The time spent on tasks were estimated using observation and staff input.

## 3. Results

### 3.1. Patterns in Historical Provider Behavior

**Figure 2:**
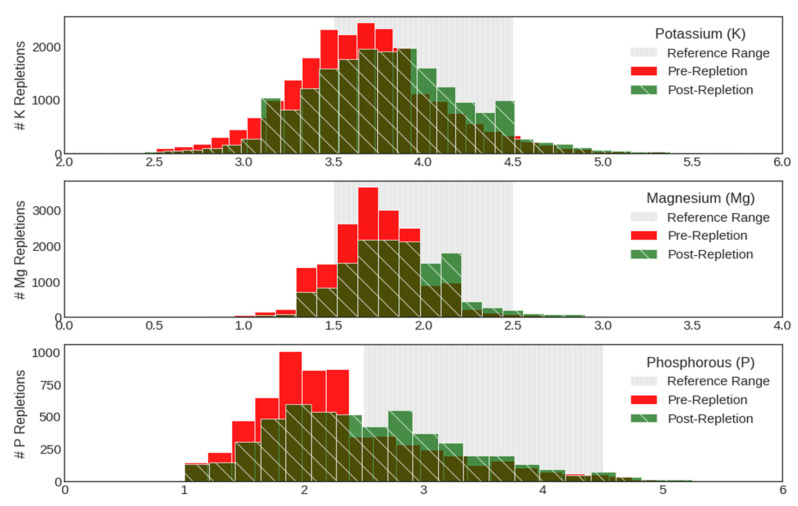
Distribution of electrolyte levels as executed by providers in historical dataset representing pre-repletion (red) and post-repletion events (green), along with the target range of electrolyte levels, in gray.

In analyzing repletion patterns in terms of the distribution of pre-and post-repletion electrolyte measurements, we found that the large majority (73% potassium, 88% magnesium, and 38% phosphate) of replacements were ordered while electrolyte levels were either within or above the reference range (Figure 2). In fact, potassium and magnesium were over-repleted at a rate of 4.4% and 1.4%. Phosphate was rarely over-treated by comparison, with just 0.6% of repletion events occurring above the target phosphate range. In addition, replacement at low electrolyte levels often failed to bring post-repletion values into the reference range (Figure 2).

### 3.2. AI-Driven Repletion Recommendations

We used inverse reinforcement learning (IRL, Appendix A), to estimate the relative weights in the reward function of each of four variables—IV repletion cost, PO repletion cost, abnormally high, and abnormally low electrolyte values—for historical UPHS data in the case of potassium (K) and magnesium (Mg). Surprisingly, we estimate small negative weights on both the cost of IV and the cost of PO repletion driving historical policy (Table 3). We compare this with the same weights chosen for training an AI-driven repletion protocol and demonstrate that this represents a substantial shift in weights relative to historical behavior, suggesting a more cost-aware repletion protocol (Table 3).

**Figure 3:**
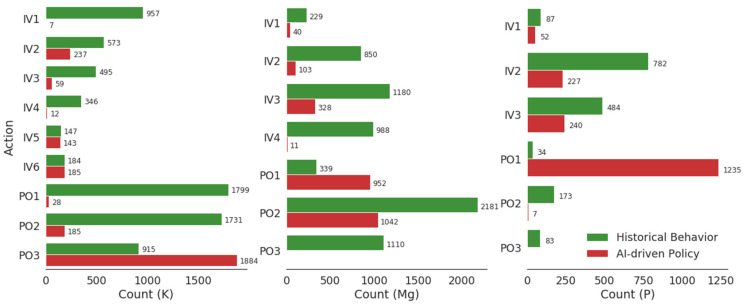
Distribution of repletion dosage levels chosen for three electrolytes in the historical data (UPHS) vs. dosages recommended by the learned RL policy.

Consequently, the learned RL protocol using this IRL-learned reward function led to policies that recommended less frequent repletion in the case of potassium and magnesium, with reductions of 61.7% and 63.9%, respectively (Figure 3). The RL-based system also showed a preference towards orally administered repletion for all three electrolytes considered, with higher doses of oral potassium replacement and higher doses of intravenous repletion for all three electrolytes when this route was chosen by the system. Compared to historical data, instances of intravenous potassium replacement dropped by 75% and oral replacement dropped by 50% (Figure 3).

**Figure 4:**
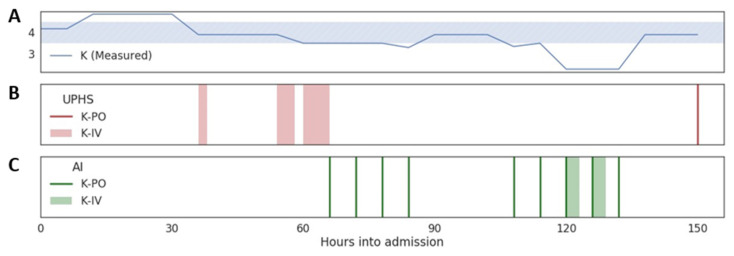
Panel (**A**) captures measured potassium (*y*-axis) across hours into patient admission (*x*-axis) with the gray ribbon visualizing the optimal range of potassium. Panel (**B**) is potassium repletion as performed by provider in historical data across hours into patient admission. Panel (**C**) is the recommendation for repletion across hours into patient admission driven by the learned RL protocol. The length of the shaded K-IV events indicates duration of infusion time.

Our optimal policy recommended repletion only when potassium was below the threshold of the reference range, and intravenous replacement only when the patient was significantly hypokalemic (data not shown). We can study the learned policy for a single patient visit to explain the behavior of the policy. The learned policy recommends fewer replacement interventions when electrolyte level is normal and more frequent repletion when the patient’s electrolyte level is low (Figure 4). The AI-driven protocol favored K-PO (oral repletion), recommending K-IV (intravenous repletion) only when potassium levels were far below the reference range, and also tended to recommend repletion more promptly following a hypokalemic event. 

**Figure 5:**
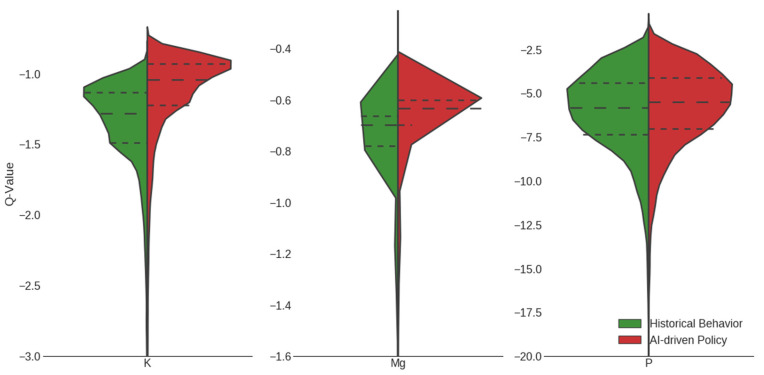
Estimated performance of policy for potassium (K), magnesium (Mg), and phosphate (P) measured by the *Q*-value prediction, which corresponds to the expected total rewards (time saved, money saved, avoidance of near misses, and side effects) during the entire patient admission. For all three electrolyte policies, the mean Q-value prediction of state–action pairs in the test set was higher for the learned RL policy than for clinician behavior observed in the UPHS data. This suggests that RL optimizes the reward function to create a learned policy that is better than clinician behavior.

In order to quantify how our system compares with the performance of historical behavior with respect to our weighted reward function, we used Fitted-Q evaluation (FQE). The *Q*-value provides a measure of policy effectiveness. Plotting the distribution of values (that is, expected accumulated rewards) for the set of all pairs of patient states and actions in the data, we found that the average reward for the learned RL protocol was higher than that for the historical data in the case of all three electrolyte policies (Figure 5). This difference is especially pronounced in the case of potassium and magnesium, emphasizing the scope of possible improvement in current practice with respect to electrolyte repletion.

### 3.3. Expected Outcomes of Implementing AI-Driven Protocol

We compared the repletion events in the historical data for the 6164 patients in our test set with the instances of recommended repletion according to our learned RL policy, accounting for both the shift towards oral repletion and the overall reduction in repletion events. We calculated the potential decrease in the cost of medication over the full five-year period to be from USD 62k to USD 20.5k. The corresponding estimated expenses related to customary lab work were reduced from USD 87.2k to USD 38k. When we included expenses related to the time spent by different healthcare providers with lab and drug expenses, the total expected expenditures from an RL-driven process were reduced from USD 519k to USD 156k, translating into a savings of USD 790 per hospital visit.

Beyond these direct cost savings, the RL system also avoids replacement of electrolytes when the patient is above the reference range, reducing potential harm to the patient, promoting precise electrolyte replacement, and avoiding potential misses and near misses.

### 3.4. Validation of the Protocol

**Figure 6:**
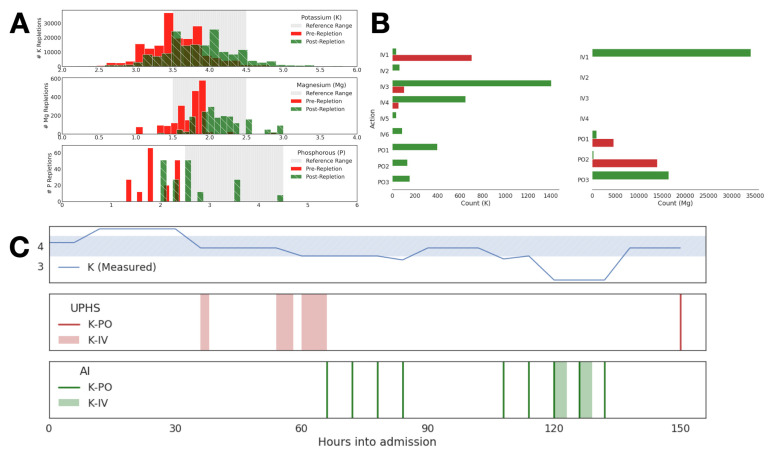
The performance of providers in the MIMIC database was similar to that observed in UPHS with frequent over-repletion (Panel (**A**)). Implementation of the RL AI-driven policy resulted in an insignificant shift in repletion patterns (Panel (**B**)), but only when the repletion was adequate (Panel (**C**)).

We validated the learned electrolyte repletion policy by testing the policy estimated from the UPHS cohort in EHR data from the MIMIC-IV cohort. The electrolyte replacement patterns in the MIMIC-IV database were similar to those observed in UPHS (Figure 6A). Similar to the UPHS test data, the application of the RL protocol learned from the UPHS cohort and applied to the MIMIC-IV cohort resulted in a shift towards PO dosages and less frequent replacement (Figure 6B). The learned RL protocol, in general, recommends repletion less frequently than reported in the MIMIC-IV dataset, reflecting the lower frequency of repletion in the UPHS data relative to the MIMIC-IV data. Finally, we confirmed that the learned RL protocol uses covariates similarly to suggest optimal actions in the MIMIC-IV dataset as in the UPHS data (Figure 6C).

## 4. Discussion

This is the first demonstration of an RL-derived treatment protocol in an ICU setting, intended to provide potentially continuous recommendations for clinician-in-the-loop patient care to address the issue of electrolyte replacements. Our RL algorithm demonstrates several important variables that guide providers to replete electrolytes for the first time. Furthermore, we demonstrated in silico that we can use a reinforcement learning (RL) strategy to create a policy that differs from clinical recommendations and that uses patient characteristics at a given time and a dynamic set of clinical variables to tailor treatment to specific patient needs. Finally, RL performed similarly in datasets from two different institutions, showing equivalent behavior and improvements in clinician policies, and addressing the ever-important problem of AI validation.

The reinforcement learning system described in this paper uses available information from electronic health records of vital signs, lab tests, and administered drugs and procedures in order to estimate a patient-specific, provider-in-loop recommendation protocol for electrolyte repletion at six-hour intervals. This period was chosen as a reasonable time within the workflow of the intensive care unit. Recommendations are presented in an interpretable and hierarchical way in which the system first suggests whether or not a repletion is needed, along with the best route for repletion, and followed by the most appropriate dosage in the event that the clinician chooses to administer a repletion.

This is a more controlled system of prescribing electrolyte repletion, reflecting a quantitative data-driven decision-making pathway that caregivers often fail to follow if the decision-making process is provider- or protocol-driven [13]. The RL system provides flexibility in deciding what the clinical priorities should be, adapting them according to the electrolyte considered and to challenging clinical situations, such as chronic renal failure, liver failure, or severe morbidity, or to the workflows of the specific healthcare center [31]. Our approach therefore presents an adaptive framework for the delivery of care capable of minimizing harm and maximizing precision, considering the patient context.

Our optimal RL policy was able to recommend electrolyte replacements in a more targeted way [31,32]. The estimated reduction in recommended repletion events in the case of potassium and magnesium allows for considerable savings in the time spent by clinicians assessing electrolyte levels and the costs incurred from unnecessary or repeat orders placed without thorough re-evaluation of clinical need [1,7]. Moreover, the recommendation of electrolyte administration at pre-repletion values above the reference range is rarely if ever observed [16], eliminating potential risk to patients due to over-treatment that was observed in the historical patient data.

In addition, by placing larger penalties on intravenous rather than oral potassium repletion, we were able to arrive at a policy that chooses oral replacement where possible [32,39]. The higher effective cost of IV repletion can be justified in a number of ways: in the cost of the prescription itself of intravenous delivery, in the provider time taken to initiate and monitor the delivery of the drug, in the increased risk of overcorrection when setting the infusion rate as well as bruising, clotting, or infection at the infusion site, discomfort or infection at the infusion site, and the risk of accidental overdosing [20,39].

It is important to note that the estimates of efficacy presented here are based on retrospective evaluation, which is challenging for AI systems that use reinforcement learning with batch data. In this scenario, we do not have the ground truth as to the best possible actions to learn from, and we cannot collect additional data following our estimated policy, as in reinforcement learning for robotics or games. Furthermore, we are not able to accurately simulate this data, given the complexity of patient health trajectories. As soon as an action is taken in the historical test data that deviates from the optimal learned policy, the patient trajectory under the optimal policy decision and all subsequent treatment decisions are no longer perfectly known [8,28,31].

It can also be challenging in retrospective studies to disentangle potential confounders in the patient attributes used to determine the necessity of repletion, and care is needed to ensure that the drivers of repletion are appropriately interpreted. For example, it was observed that high serum creatinine levels increase the probability of recommending potassium repletion, assuming the patient has experienced kidney failure, resulting in the buildup of creatinine levels, and thus the need for dialysis, which in turn is likely to result in potassium deficiency. This recommendation may not hold if dialysis is not initiated or continued by the care provider. Finally, the system here focused on data between 2010 and 2015; it is possible that there has been a shift in electrolyte testing and ordering practices during or after this timeframe. The training dataset is limited to one center. We also limited the dataset to instances where data were complete, resulting in the substantial attrition of the dataset. It is unclear if this strategy provides a more robust treatment policy than using a more sizable but incomplete dataset. Further validation is needed to ensure that the repletion policy recommended is robust for this shift in time. Future developments will include the prospective validation of optimal RL policy recommendations by first running real-time side-by-side comparisons of system recommendations with providers’ actions (i.e., shadowing providers), and then evaluating the efficacy of bedside policy recommendations in a provider-in-the-loop protocol.

Developing this data-driven decision support tool is one task, but its implementation into a clinical workflow may also encounter several obstacles. Providers may mistrust the automated recommendations, in particular where there is a substantial departure from current practice. This may occur, for instance, when providers are inclined to frequent recommendations of higher doses of PO repletion. In addition, questions of reimbursement, liability, and accountability may arise, and hospital systems need to figure out how to deal with operational and legal consequences of implementation [12,41]. However, the potential gains of thoughtful, well-planned implementation are considerable. Our estimation of the financial benefit is conservative and does not account for other factors that could not be quantified in the data [22,23].

The next step of this project is to develop an easily implemented module allowing for processing data from various healthcare systems to provide more cross-validation to assess the robustness of the algorithm against regional differences and more systemic biases related to practice patterns and biases. The implementation of the RL will be challenging, and one way to design the algorithm is to allow it to advise physicians during patient rounds. Designing the RL to work in a six-hour interval was carried out with that idea in mind. Because the RL algorithm is able to integrate new data into the optimal policy, these adaptive policies are uniquely suited for robust deployment in a variety of environments.

In summary, this work describes an approach to guiding the repletion of electrolytes of patients in the ICU, with the aim of avoiding the need for the patient to undergo prolonged durations of electrolyte imbalance, while minimizing the costs associated with ordering and administering oral and intravenous repletion.

## Figures and Tables

**Table 1:** Selected 52 clinical features from patient EHRs based on their influence on electrolyte levels. We also included imputed measurements at each 6 h interval for a number of key vitals and labs.

	Features
Static	Age, Gender, Weight, Floor/ICU
Vitals	Heart rate, Respiratory rate, Temperature, O_2_ saturation pulse oximetry (SpO_2_), Urine output, Non-invasive blood pressure (systolic, diastolic)
Labs—Raw	K, Mg, P, Ma, Chloride, Anion gap, Creatinine, Hemoglobin, Glucose, Blood Urea Nitrogen, WBC Count
Labs—Indicator	Ca (Ionized), Glucose, CPK, LDH, ALT, AST, PTH
Drugs	K-IV, K-PO, Mg-IV, Mg-PO, P-IV, P-PO, Ca-IV, Ca-PO, Loop diuretics, Thiazides, Acetazolamide, Spironolactone, Fluids, Vasopressors, β-blockers, Ca-blockers, Dextrose, Insulin, Kayexalate, TPN, PN, PO nutrition
Procedures	Packed-cell transfusion, Dialysis

**Table 2:** Repletion of K, Mg, and P replacements in terms of dose and duration.

		Oral (PO)			Intravenous (IV)					
		PO1	PO2	PO3	IV1	IV2	IV3	IV4	IV5	IV6
*K*	0	20 mg	40 mg	60 mg	20 mEq 2 h	40 mEq 4 h	60 mEq 6 h	20 mEq 1 h	40 mEq 2 h	60 mEq 3 h
*Mg*	0	400 mg	800 mg	1200 mg	0.5 g 1 h	1 g 1 h	1 g 2 h	1 g 3 h		
*P*	0	250 mg	500 mg	750 mg	15 mEq 1 h	30 mEq 3 h	45 mEq 6 h			

**Table 3:** Weights of four variables driving electrolyte repletion (IV repletion cost, PO cost, abnormally high, and abnormally low electrolyte values) in the historical dataset and after application of reinforcement learning (RL) algorithm showed substantial changes.

	Historical Policy Drivers	AI Policy Drivers
*K*	(−0.05, −0.08, 0.20, 0.67)	(0.07, 0.04, 0.15, 0.74)
*Mg*	(−0.05, −0.01, 0.33, 0.61)	(0.01, 0.01, 0.48, 0.48)
*P*	(−0.25, 0.11, 0.30, 0.34)	(0.08, 0.07, 0.5, 0.35)

**Figure A1:**
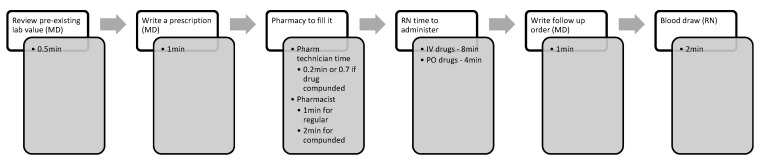
The workflow used for estimation of time and costs related to savings after the introduction of RL to a clinical setting as a clinician-in-the-loop decision-making support tool.

## Data Availability

The datasets used and/or analyzed during the current study are available from the corresponding authors on reasonable request upon approval from the IRB.

## Appendix A

### Appendix A.1. Reward Design

The overall reward function can be written as *R = w × ϕ*, where *ϕ(s,a,s′)* is a four-dimensional vector function parameterized by the current state *s*, immediate action *a*, and next state *s′* that formalizes each of objectives described, and *w* defines the relative weight of each of these objectives. Penalties for values above and below the reference range are applied independently to allow for asymmetric weighting of the risks posed by hypokalemia when compared with hyperkalemia. A sigmoid function to model penalties on abnormal vitals reflects the clinical importance of a more severe electrolyte imbalance.

Vector functions *ϕ* for both magnesium and phosphate are also needed, with elements corresponding to IV repletion cost, PO repletion cost, and abnormally high and abnormal low electrolyte levels.

$$\phi_{t}\left(\cdot \right)=\left[\begin{array}{c} -1\left[a_{t}^{route}\left[0\right]\right]\\ -1\left[a_{t}^{route}\left[1\right]\right]\\ -1\left[s_{t+1}\left[K\right]>K_{max}\right]\cdot 10*(1+\exp{\left(-\sigma \left(K-K_{max}-1\right)\right)}^{-1}\\ -1\left[s_{t+1}\left[K\right]<K_{min}\right]\cdot 10*(1-\exp{\left(-\sigma \left(K-K_{min}+1\right)\right)}^{-1} \end{array}\right]\in \left[\begin{array}{c} 0,-1\\ 0,-1\\ \left(-10,0\right)\\ \left(-10,0\right) \end{array}\right]$$

In both the UPHS and MIMIC datasets, we used a 75–25 training–test split. We used the two datasets to model this clinical decision-making problem as a Markov decision process (MDP) and used a custom-designed reward function that penalizes states in which the patient is outside the given reference range for an electrolyte. We then used batch FQI to learn an optimal policy and find that our learned Q-table converges (i.e., stabilizes) after 25–50 iterations [30].

### Appendix A.2. Fitted Q Iteration (FQI)

The FQI algorithm learns an estimator for value Q of each state–action pair in our MDP, where Q is the expected discounted cumulative reward, starting from the given state and taking the specified action. This algorithm uses a series of regression models, where the target Q-values for the regression at each iteration are obtained by bootstrapping on the estimated Q from the previous regression, and updating based on observed rewards in the current iteration [34,35]. FQI offers flexibility in the use of any regression method to solve the supervised problems at each iteration. We fitted our estimate of Q at each iteration of FQI, using gradient boosting machines (GBMs) [32]. This is an ensemble method in which weaker predictive models, such as decision trees, are built sequentially by training on residual errors, thereby allowing models to learn higher-order terms and more complex interactions amongst features [33].

### Appendix A.3. Inverse Reinforcement Learning

Inverse reinforcement learning is the task of extracting the reward function, which explains the observed behavior in the data. In this case, it involves determining the value of reward weights *w* where *R = w × ϕ* gives us an optimal policy—similar to the policy followed by clinicians in the past. This is typically carried out by arbitrarily choosing initial weights *w*, solving for a policy that optimizes reward *R = w × ϕ*, estimating some representation of the dynamics of this policy, comparing the policy dynamics with the behavior seen in historical data, and updating weights accordingly, then iterating until the learned policy with our weights is acceptably close to past behavior. In this case, we first set *w* to assign equal priority to all objectives in *ϕ* and used the discounted time spent in each state to represent policy dynamics, using this as update *w*.
